# Advanced oxidation processes at water/hydrophobic interfaces: energy-fluctuation mechanism and electron utilization quantification

**DOI:** 10.1039/d5sc08827e

**Published:** 2026-02-03

**Authors:** Gaobo Xu, Fuling Li, Jin Ye, Shujun Zhang, Haiqin Ma, Guangdong Zhou, Cunyun Xu, Xiaofeng He, Xiude Yang, Qunliang Song

**Affiliations:** a Institute for Clean Energy and Advanced Materials, School of Materials and Energy, Southwest University Chongqing 400715 P. R. China qlsong@swu.edu.cn; b Shanghai Institute of Microsystem and Information Technology, Chinese Academy of Sciences Shanghai 200050 P. R. China; c School of Physics and Electronic Science, Zunyi Normal College Zunyi 563002 P. R. China

## Abstract

The fundamental driving force and mechanism of water/hydrophobic interface chemistry remain debated. Contact-electro-catalysis (CEC), which converts mechanical energy into extensive interfacial charge separation in water, has introduced a new perspective. However, the introduction of ultrasonication has prompted a renewed scrutiny of its reaction mechanisms. At the same time, those studies have no quantification assessment due to the calculation difficulty of energy-to-electron conversion. Here, we investigate radical-mediated advanced oxidation processes (AOPs), at a macroscopic water/hydrophobic interface without violent energy input. Theoretical analysis reveals that the flexoelectric response of interfacial water creates a local polarization field that is strong enough to separate electrons from H_2_O or OH^−^. These interfacial energy fluctuations are thus proposed as the primary origin of the reaction driving force. Furthermore, by leveraging a quantifiable press-and-release device, we establish a methodological framework for evaluating the triboelectric electron utilization ratio in CEC, yielding a first estimation of ∼44.8%. This work provides new insights into both interfacial AOPs and contact electrification at water/hydrophobic interfaces. This breakthrough offers a new and sustainable strategy for low-energy water purification and pollutant degradation, and also provides a basis for future precise quantification of electron utilization efficiency.

## Abstract 2

AOPs, traditionally limited to microscopic interfaces, are extended to a macroscopic water/hydrophobic interface without intense energy input. Flexoelectricity couples interfacial energy fluctuations with the energy required for charge transfer.

## Graphical abstract

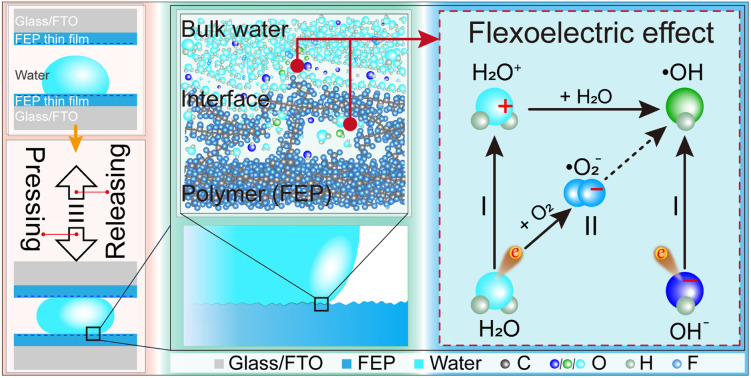

## Introduction

Water, one of the most abundant substances on Earth's surface, sustains all life. Interfaces formed between two different phases, most often involving water, serve as the privileged locations for a variety of physical,^1^ chemical,^2^ and biological^3^ processes. By reducing the size and incurving water or another phase that can form interfaces with water, aerosol microdroplets of water,^4,5^ microbubbles in water,^6^ oil–water emulsions,^7^ or polymer micro particles in water^8^ can be produced. A defining feature shared by these systems is the so-called “water/hydrophobic” micro-interface,^9^ with the less understood hydrophobic force. Reducing down to microscale water/hydrophobic contacts, these interfaces impart several distinctive properties to interfacial water, including accelerated water evaporation,^10^ interfacial solvation,^11^ extreme pH environments,^12,13^ molecular rearrangement,^14^ and spontaneously generated high electric fields. These unusual characteristics have enabled advanced oxidation processes (AOPs), which never occurred in pure bulk water but have been identified and/or vastly accelerated at such micro-interfaces, providing a chance for clean chemical synthesis and organic degradation environmentally.^9,14^ However, the complex coupling of these interfacial properties poses fundamental challenges to mechanistic understanding, particularly regarding the origin of the driving force (energy) that initiates these interfacial reactions. Even the locations at which these specific reactions occur are still under debate. Violent energy inputs such as electrospray ionization^15^ to prepare microdroplets with excess charges, gas nebulization^16^ or ultrasonic cavitation^17^ to obtain fine mist having reactive radicals, or ultrasonic vibration^18^ to realize the contact-separation between water and polymer microparticles, open an alternative explanation of the observed reactions. That is, the reaction might happen in bulk instead of water/hydrophobic interfaces,^19,20^ diminishing the importance of water/hydrophobic interfaces. Nonviolent preparation methods like water condensation^21^ or levitation,^22^ and very recent unactivated microdroplets obtained by adiabatic expansion or placing dry ice in water^23^ conclusively emphasize the role of water/hydrophobic interfaces in the mysterious droplet chemistry. However, a reasonable explanation for the original source (energy) driving AOPs at the water/hydrophobic interface has not yet been provided. Due to the common importance of water/hydrophobic interfaces and the non-diminishing character of polymer microparticles in water, interfaces formed with a large volume of water and polymer microparticles have attracted considerable attention.

**Scheme 1:**
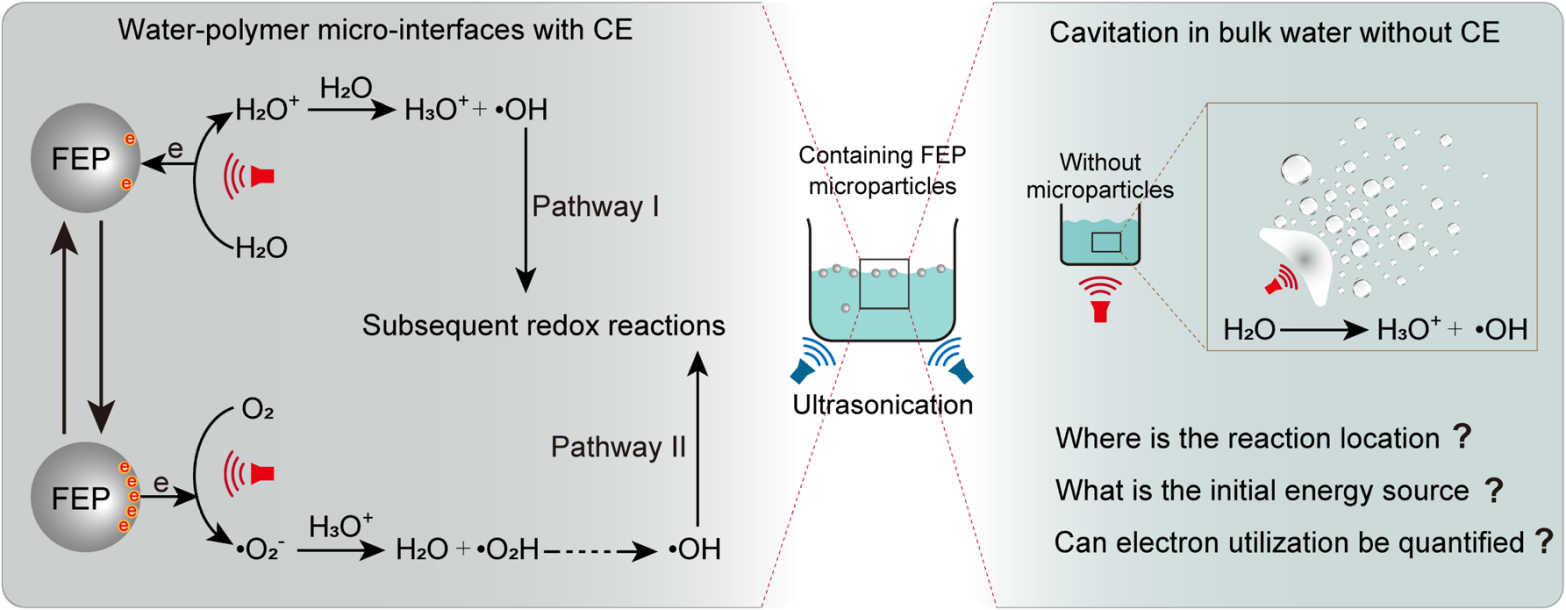
Reaction mechanism at the water/hydrophobic micro-interface driven by ultrasound (left) and possible reactions occurring in bulk water under intense energy input (right).

Recently, AOPs catalyzed by the contact electrification (CE) between water and hydrophobic polymer microparticles have been reported,^8,24^ a phenomenon referred to as contact-electro-catalysis (CEC). In this CEC process, CE converts the kinetic energy of water and microparticles into extensive charge separations at the water/hydrophobic micro-interface, as shown in Scheme 1 (left). From this perspective, CE may represent the original source that drives interfacial AOPs, despite ongoing debates regarding its mechanism.^9,24,25^ However, CEC is typically driven by vigorous external energy inputs, such as ultrasonication^8^ or ball milling,^26^ because sufficient energy is required to release the catalyst surface-trapped electrons.^27^ Similar to other water/hydrophobic interfaces with violent energy inputs,^9^ this may well return to the original questions that whether the observed catalysis occurs at the water/hydrophobic micro-interface or in the bulk phase, as shown in Scheme 1 (right). To elucidate the CEC mechanism, especially the energy source, for the observed AOPs happening at water/hydrophobic interfaces, vigorous energy inputs should be avoided while preserving the water–polymer interfaces. Meanwhile, due to the difficulty in quantifying the total number of electrons generated through energy-to-electron conversion in ultrasound-driven CEC systems, current studies have not yet carried out further assessments of electron utilization efficiency.

In this study, we adopted a mild energy input strategy based on gentle press-and-release cycles to avoid induced bulk reactions, using macroscale water/hydrophobic interfaces formed by a large volume of water and a bulk hydrophobic polymer thin film to replace the microscale interfaces. The macroscopic water/hydrophobic interface AOP system driven by mild energy input also decouples the complex interplay of enigmatic phenomena induced by the aforementioned micro-size effects. The advantage of easily observable chemical reactions due to the vast number of microdroplets, which provides a large number of reaction events, can be compensated by increasing the contact-separation cycles of macroscale water/hydrophobic interfaces.

Experimental results demonstrate that, even in the absence of an intense energy input, AOPs resembling those previously reported at water/hydrophobic micro-interfaces were observed at the macroscale water/hydrophobic interface. Significant generation of hydroxyl radicals (˙OH) was detected at the dynamic macroscopic interface. Water/hydrophobic interfacial phenomena, including the excessive accumulation of OH^−^ ions, the directional reorientation of the hydrogen-bond network, and the delocalized distribution of water molecules on hydrophobic polymer surfaces, collectively provide numerous sources for the generation of ˙OH. The flexoelectric effect (Explanation of Key Concepts in the SI), which has been reported to give rise to flexoelectric potential differences on the order of, or even exceeding, 10^9^ V m^−1^,^28–30^ is expected to be triggered within the first few layers of interfacial water during the periodic construction and destruction of the water/hydrophobic interface. Structure fluctuations and the resulting energy fluctuations in interfacial water induced by the dynamic water/hydrophobic interface during the pressing-and-releasing motion thus may directly account for the generation of flexoelectric polarization fields. This is believed to serve as the initial energy source for ˙OH generation, by extracting electrons either from OH^−^ ions or directly from water molecules situated at the interface. It is worth emphasizing that, thanks to the triboelectric nanogenerator (TENG) characteristics of the macroscale water/hydrophobic interface system driven by mild mechanical energy, we can directly measure the amount of charge transferred during operation. By experimentally determining the charge consumed during MO degradation, we further performed a preliminary estimation of the electron utilization in the CEC reaction, which is approximately 44.8%. Such quantification is difficult to achieve in ultrasonically driven CEC systems, as the total number of electrons generated in those systems cannot be readily determined.

Our work experimentally demonstrates and systematically elucidates the chemical reaction process at the macroscale water/hydrophobic interface without intense energy input. The proposed driving force (structure fluctuation induced energy fluctuations) for charge transfer provides guidance and new perspectives for further exploration of either CE or chemical reactions at water/hydrophobic interfaces. Meanwhile, it offers a feasible strategy for quantifying electron utilization efficiency in CEC.

## Experimental

### Materials

Unless otherwise specified, all chemicals used in this study were commercially available and used without further purification. Methyl orange (MO, C_14_H_14_N_3_NaO_3_S, 98%) and *tert*-butyl alcohol (TBA, C_4_H_10_O, >99.5%) were purchased from Shanghai Macklin Biochemical Technology Co., Ltd. 5,5-Dimethyl-1-pyrroline *N*-oxide (DMPO, C_6_H_11_NO, ≥99%) and dimethyl sulfoxide (DMSO, C_2_H_6_OS, >99.9%) were obtained from Sigma-Aldrich (Shanghai) Trading Co., Ltd. Silver nitrate (AgNO_3_, ≥99.8%) was purchased from Chron Chemicals Co., Ltd. Fluorinated ethylene propylene thin films (FEP, 80 µm thick) were obtained from Taizhou Chenguang Plastic Co., Ltd. Fluorine-doped tin oxide (FTO) glass substrates were purchased from Liaoning Optimal New Energy Technology Co., Ltd. High-purity oxygen and argon gases were supplied by Chongqing Xingye Gas Co., Ltd. Deionized water (resistivity: 18.2 MΩ·cm) was prepared using a PURELAB Option-Q 7/15 ultrapure water system. Compressed air with a relative humidity of approximately 10% was supplied by a self-built air compression system. During operation, water or aqueous solution was placed between the upper and lower friction layers, and the periodic actuation of the linear motor reproducibly generated water–hydrophobic interfaces.

### Experimental setup

To eliminate surface contaminants and prevent potential damage to the FEP film during subsequent steps, the glass/FTO substrates were first cleaned by ultrasonication. An 80 µm-thick FEP thin film was then adhered onto the dried glass/FTO substrate, ensuring no air bubbles were introduced. The FEP hydrophobic surface of the lower glass/FTO/FEP assembly was subjected to patterned plasma etching (45 W, 180 s, air as the working gas) using a stainless-steel mask with a 3 mm aperture. Two glass/FTO/FEP assemblies were placed face to face, with their FEP surfaces aligned opposite to each other. The lower substrate was positioned on a flat platform as the stator and the upper one was fixed to a vertically mounted miniature linear motor as the mover. To avoid excessive compression between the two substrates, a ∼1.5 mm thick soft sponge was placed beneath the lower substrate as a cushion layer.

### Characterization

A high-speed camera (Huaray, MV-A5031MU 815) was used to record the motion of large-volume droplets between the upper and lower hydrophobic layers. Electron paramagnetic resonance (EPR, Bruker EMXplus X-band) spectroscopy was employed to detect radical species generated at the water–hydrophobic interface. UV–vis absorption spectra of MO aqueous solutions in the 200–700 nm range were recorded using a UV–vis spectrophotometer (PerkinElmer Lambda 365+). A non-contact electrostatic field meter (SIMCO, FMX-004) was used to measure the surface potential of charged FEP thin films. The hydrophobicity and surface morphology of FEP thin films before and after plasma etching were evaluated using a contact angle goniometer (POWEREACH, Shanghai Zhongchen Digital Technology) and a scanning electron microscope (SEM, JEOL JSM-7800F), respectively. X-ray photoelectron spectroscopy (XPS, Shimadzu AXIS Supra+) and Fourier-transform infrared (FTIR, PerkinElmer Spectrum Two) spectroscopy were employed to analyze the elemental and functional group distributions on the FEP surface before and after the reaction, respectively. The transferred charge was measured using an electrometer (Keithley 6514) coupled with a data acquisition card (NI PCI-6259).

### Atmosphere control

A semi-enclosed chamber was fabricated using 5 mm-thick acrylic plates to regulate the reaction environment. Detailed configurations for atmospheric control are shown in Fig. S3 with the corresponding descriptions.

## Results and discussion

### Macroscale water/hydrophobic interfaces under mild energy input

**Fig. 1:**
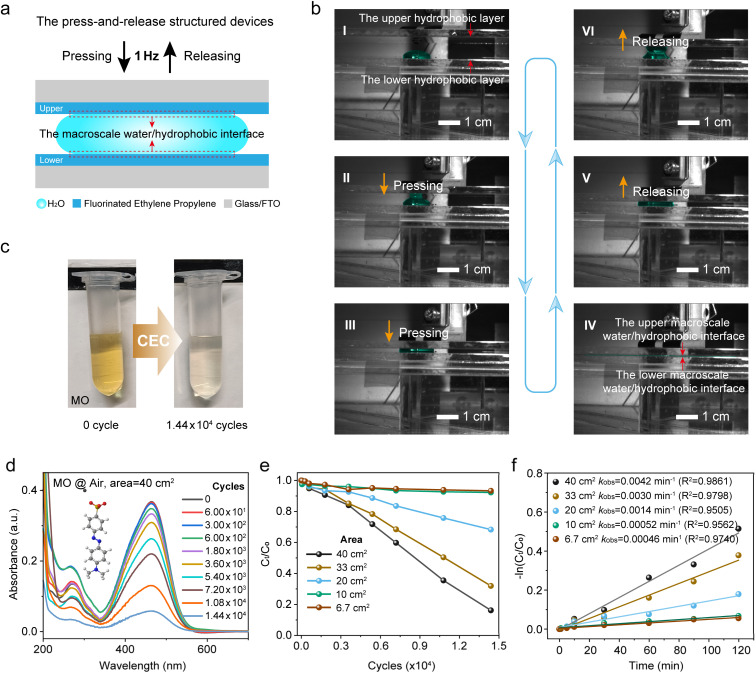
(a and b) Schematic illustration and optical photograph of the macroscopic water/hydrophobic interface. (c) Photographs showing the color change of the MO solution after 1.44 × 10^4^ press-and-release cycles at 1 Hz. (d) UV–vis spectra of the MO solution recorded during the press-and-release cycles. (e) Absorbance of the MO solution at 465 nm under different droplet spreading areas. (f) Pseudo-first-order degradation rate constants of the MO solution at varying droplet spreading areas.

Building on previous work on droplet electricity generators (DEGs) for harvesting electricity,^31–33^ the developed press-and-release structure was adopted to effectively regulate the contact and separation between a large volume droplet and triboelectric materials, allowing the facile and reversible formation of macroscale water/hydrophobic interfaces. In this study, commercial bulk fluorinated ethylene propylene (FEP) thin films and FTO-coated glass substrates were selected to form a glass/FTO/FEP – water or aqueous solution – FEP/FTO/glass configuration (Fig. 1a and S1a). As shown in Fig. 1a, the FEP serves as the hydrophobic layer, and the contact between the spread water and FEP constitutes the macroscale water/hydrophobic interface. A low-frequency mechanical force (1 Hz, significantly lower than the typical tens to hundreds of kHz of ultrasonic vibration^8^) was applied, solely to alter the droplet morphology and thereby control the water/hydrophobic interface. Spacers positioned at the four corners provided ample and stable space, allowing the droplet to spread freely between the hydrophobic layers (Fig. S1b). The droplet spreads under compression between the upper and lower hydrophobic layers, retracts due to surface tension as the layers relax, as captured in high-speed imaging (Fig. 1b). Here, stages i−iii correspond to the pressing process, which leads to the fully compressed state at stage iv, where the largest macroscopic water/hydrophobic interface is formed. This is followed by stages v−vi, representing the release process, during which the established interface is disrupted. A complete press-and-release cycle, as described above, takes 1 second. To consistently center the droplet within the hydrophobic layers, a localized plasma etching treatment was performed at the central of the lower hydrophobic FEP (Fig. S2). Another critical design consideration was the incorporation of a back FTO electrode, which effectively screens the surface charges on the bare FEP thin film and suppresses electrostatic discharge at the liquid-solid–gas triple-phase interfaces.^34^ Unless stated otherwise, all experiments were conducted under an air atmosphere at room temperature.

Given the absence of any form of intense energy input, such as shock waves, cavitation, or other high-frequency mechanical forces, and the deliberate suppression of potential discharge phenomena at the triple-phase contact line, the macroscale water/hydrophobic interface introduced in this experiment can be considered unaffected by possible bulk-phase reactions or the enigmatic characteristics typically associated with microdroplets. This allows the reaction to be focused exclusively on the water/hydrophobic interface. To investigate AOPs happening at the water/hydrophobic interface, an aqueous solution of methyl orange (MO), commonly used as a reaction indicator, was employed to verify the reaction process. A sealed, atmosphere-controlled chamber was constructed to regulate the macroscopic water/hydrophobic interface environment, where all primary reactions in this study were carried out (Fig. S3).

### AOPs observed at the macroscopic water/hydrophobic interface

Another reason for choosing MO as the reaction indicator is its anionic nature, which matches the negative surface charge of FEP. This feature effectively suppresses physical adsorption of MO onto FEP, thereby minimizing potential interference with the accurate assessment of the interfacial reaction. As shown in Fig. 1c, the original MO aqueous solution (5 ppm) exhibited a visually discernible discoloration after 1.44 × 10^4^ press-and-release cycles. To further quantify this observation, ultraviolet-visible (UV–vis) spectroscopy was employed to monitor the absorption spectra of the MO solution at different press-and-release cycles (0, 6 × 10^1^, 3 × 10^2^, 6 × 10^2^, 1.8 × 10^3^, 3.6 × 10^3^, 5.4 × 10^3^, 7.2 × 10^3^, 1.08 × 10^4^, and 1.44 × 10^4^), with the corresponding spectra and optical photograph shown in Fig. 1d and S4, respectively. The results reveal a progressive decrease in absorbance, indicating gradual fading of the MO solution, with the concentration reduced to 16.2% after 1.44 × 10^4^ cycles. Further characterizing the FEP surface after 1.44 × 10^4^ press-and-release cycles by X-ray photoelectron spectroscopy (XPS), UV–vis spectroscopy, and Fourier transform infrared (FTIR) spectroscopy (Fig. S5−7) showed no detectable MO residues, thereby excluding physical adsorption on FEP. Given that the water layer thickness (d) directly determines the area of the water/hydrophobic interface, it is expected to have a significant impact on MO degradation. A quantitative analysis (Fig. 1e, S8 and S9) indicates that larger droplet spreading areas, which correspond to thinner water layers, facilitate faster and more efficient degradation. Specifically, after 1.44 × 10^4^ press-and-release cycles, the MO concentration decreased to 16.20%, 31.98%, 68.30%, 92.26%, and 93.33% for droplet spreading areas of 40, 33, 20, 10, and 6.7 cm^2^, respectively. As shown in Fig. 1f, the pseudo-first-order rate constants of MO degradation were obtained from linear fittings of ln(*C*_t_/*C*_0_) *versus* time, increasing from 0.00046 s^−1^ at 6.7 cm^2^ to 0.0042 s^−1^ at 40 cm^2^, indicating that larger droplet spreading areas accelerate the degradation. The fits yielded *R*^2^ (the coefficient of determination) values ranging from 0.9505 to 0.9861, demonstrating good linearity and validating the pseudo-first-order approximation. These results underscore the importance of interfacial contact area in enhancing the degradation process. In short, these results clearly demonstrate that chemical reactions associated with the discoloration of MO occur at the macroscale water/hydrophobic interface. As a control, we held the macroscale water/hydrophobic interface in a static state (Fig. S10 and S11) to further validate the behavior observed under dynamic conditions. No appreciable color change of MO was observed after 240 min without repeated press-and-release cycles, highlighting the essential role of dynamic interface construction and destruction in driving the chemical reactions.

### Generation of reactive oxygen species (ROS) and their role in oxidative reactions

**Fig. 2:**
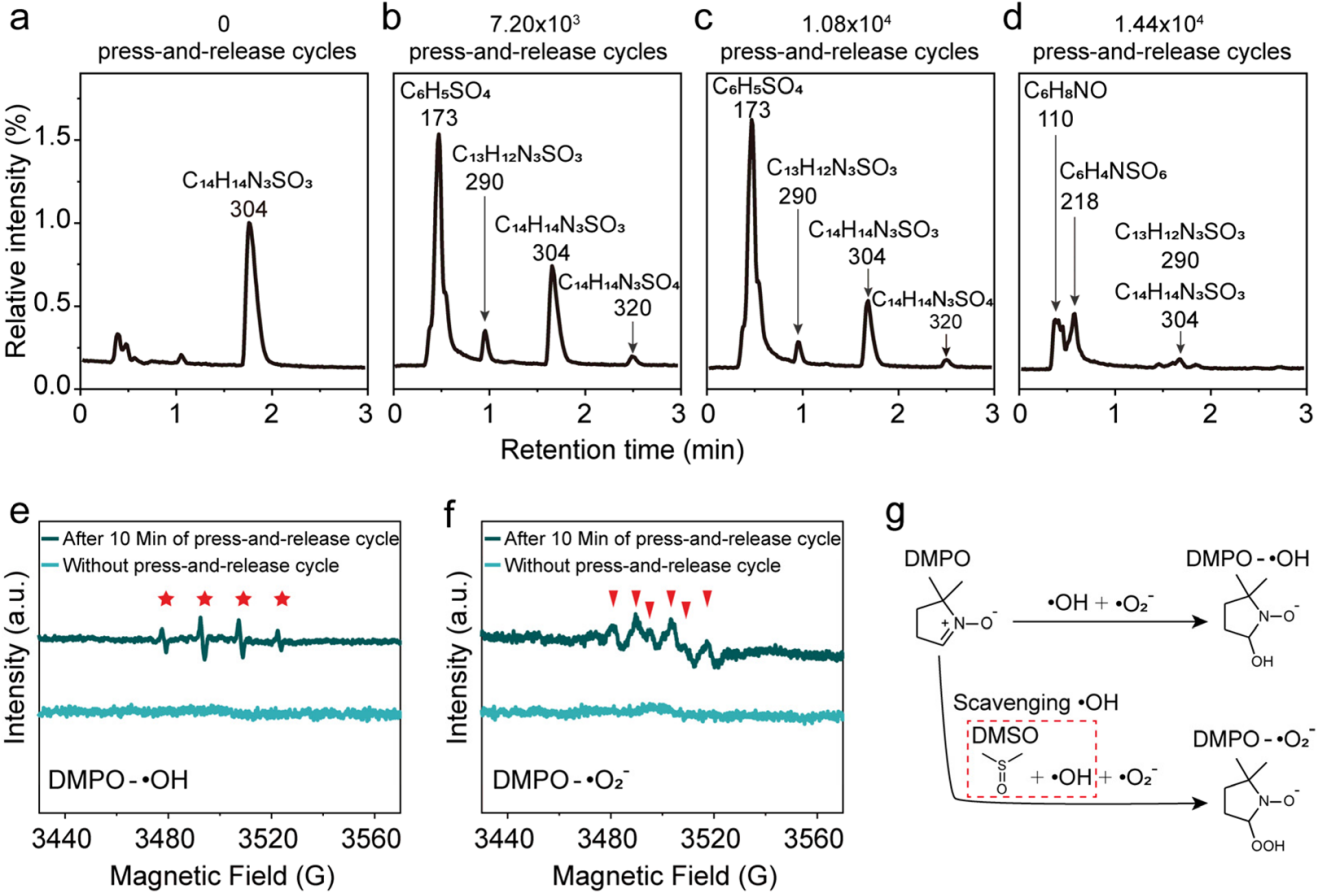
LC-MS and EPR spectra of the water solution after chemical reactions at the macroscopic water/hydrophobic interface: (a–d) LC-MS spectra of the MO solution before and after 7.2 × 10^3^, 1.08 × 10^4^, and 1.44 × 10^4^ press–release cycles, respectively. EPR results: (e) ˙OH signals and (f) ˙O_2_^−^ signals. (g) Schematic illustration of the influence of DMSO on DMPO-adduct formation.

To elucidate the degradation mechanism of MO, we systematically investigated the formation of intermediates during the interfacial reaction at the water/hydrophobic interface. MO solutions collected after various press-and-release cycling durations were analyzed using high-resolution liquid chromatography-mass spectrometry (LC-MS), as shown in Fig. 2a–d and S12–15. For clarity in comparison, the peak intensity of MO in the untreated aqueous solution was used as the normalization reference, against which the MO signal intensities of all processed samples were scaled (Fig. 2a–d). The MO signal gradually declined with increasing cycling time, indicating progressive degradation of MO at the interface. Quantitative analysis based on integrated peak areas revealed that the residual MO fractions after 7.2 × 10^3^, 1.08 × 10^4^, and 1.44 × 10^4^ cycles were 58.4%, 34.9%, and 5.94%, respectively, which is in excellent agreement with the UV–vis spectroscopy results. The mass spectra indicated the formation of several characteristic intermediate ions during MO decomposition, including C_6_H_8_NO (*m*/*z* = 110), C_6_H_5_SO_4_ (*m*/*z* = 173), C_6_H_4_NSO_6_ (*m*/*z* = 218), and C_13_H_12_N_3_SO_3_ (*m*/*z* = 290) (Fig. S12–15). Additionally, a weak signal at *m*/*z* = 320 was observed, which may originate from adducts of the parent ion with trace impurities or solvent molecules, or represent a minor byproduct formed through an alternative reaction pathway.

At water/hydrophobic micro-interfaces or in other catalytic oxidation systems, free radicals, typically hydroxyl radicals (˙OH) and superoxide anions (˙O_2_^−^), are widely recognized as key reactive intermediates.^24,35^ At macroscale water/hydrophobic interfaces without vigorous energy inputs, further evidence is needed to verify whether such radical species can be formed and participate in degradation reactions. To further address this, 5,5-dimethyl-1-pyrroline *N*-oxide (DMPO, 100 mM) was employed as a radical-trapping agent, and electron paramagnetic resonance (EPR) spectroscopy was used to detect the formation of ROS at this interface. As shown in Fig. 2e, a characteristic quartet EPR signal (1:2:2:1) corresponding to DMPO-˙OH adducts was observed after 10 min of repeated press-and-release cycles, whereas no signal was detected in control droplet without such cycling. These results demonstrate that ˙OH can be effectively generated at the macroscale water/hydrophobic interface under mild energy input. To further probe the generation of ˙O_2_^−^, dimethyl sulfoxide (DMSO) was introduced to scavenge ˙OH, thereby facilitating the trapping of ˙O_2_^−^ by DMPO (Fig. 2f and g). Distinct DMPO-˙O_2_^−^ signals were observed in the EPR spectrum after 10 min of repeated gentle press-and-release cycles, while no such signals were detected in the control samples. This suggests that molecular oxygen at the macroscale water/hydrophobic interface may capture free or quasi-free electrons to generate superoxide anions.

**Fig. 3:**
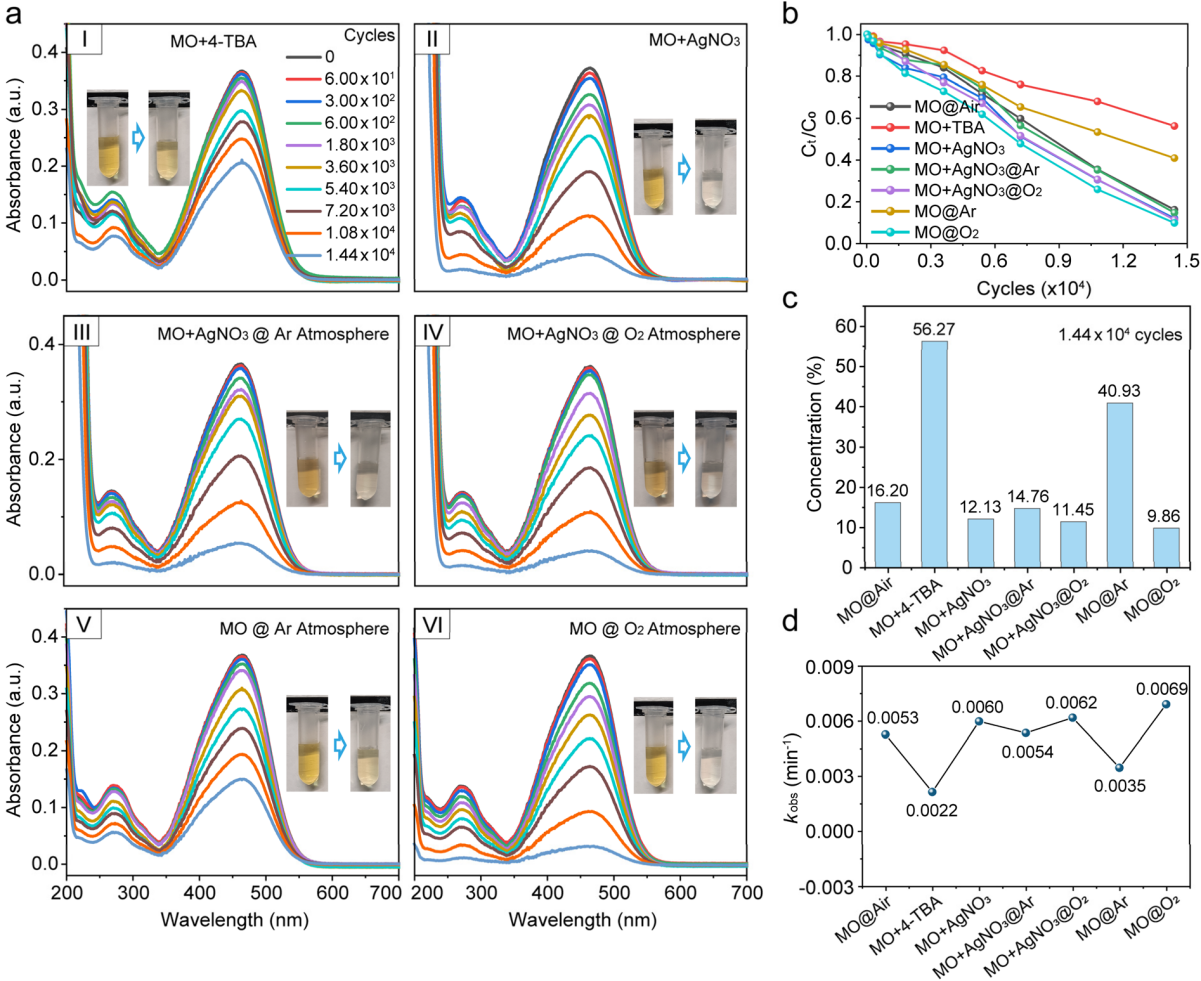
(a) UV–vis absorption spectra showing the effects of radical scavengers and atmospheric conditions on the AOPs at the water/hydrophobic interface. (b) Absorbance at 465 nm during press-and-release cycling over time under different conditions. (c) Residual concentrations of MO after 1.44 × 10^4^ press-and-release cycles under various conditions. (d) Pseudo-first-order degradation rate constants of the MO solution.

To further explore the precise contribution of reactive radicals in the macroscale water/hydrophobic interfacial reaction, 4-*tert*-butylphenol (4-TBA, 1 mM) and silver nitrate (AgNO_3_, 1 mM) were used as ˙OH and electron scavengers, respectively, as shown in Fig. 3a(I–IV), S16 and S17. The results showed that MO degradation was markedly inhibited upon ˙OH scavenging, with MO concentration dropping to merely 56.27% after 1.44 × 10^4^ press-and-release cycles (Fig. 3a(I), b and c). Notably, electron capture did not suppress the degradation as reported.^8,18^ Instead, it slightly accelerated the degradation process, with the MO concentration decreasing to 12.13% after 1.44 × 10^4^ press-and-release cycles (Fig. 3a(II), b and c). This suggests a novel free radical-driven AOP at the macroscale water/hydrophobic interface. To validate this hypothesis, we examined the influence of electron capture on MO degradation at the water/hydrophobic interface under pure O_2_ or Ar (oxygen-free) conditions, as shown in Fig. 3a(III–IV), S17 and S18. Under pure Ar, electron capture by AgNO_3_ accelerated the degradation process, reducing the MO concentration to 14.76% after 1.44 × 10^4^ press-and-release cycles (Fig. 3a(III), b and c), significantly faster than that without electron capture (40.93%, Fig. 3a(V), b and c), highlighting the role of electron capture in the interfacial AOPs. It should be noted that the inert Ar atmosphere effectively eliminates potential interference from atmospheric oxidants (*e.g.*, O_2_, O_3_, and NO_*x*_), thereby underscoring the intrinsic contribution of the macroscopic water/hydrophobic interface to the reaction. Under pure O_2_, electron capture slightly suppressed the interfacial reaction, yielding an MO concentration of 11.45% after 1.44 × 10^4^ press-and-release cycles (Fig. 3a(IV), b and c) with a marginally slowed down reaction compared to the non-electron-capture scenario (9.86%, Fig. 3a(VI), b and c), suggesting that electron capture affected the reaction pathway under oxygen-rich conditions. According to studies on the electrodynamics of water molecules,^36,37^ water dissociation produces water cation holes (H_2_O^+^) and electrons, and proton transfer and electron relaxation on the picosecond timescales, generating hydrated protons, ˙OH, and solvated electrons (hydrated electrons). Among these species, ˙OH directly engages in the interfacial AOPs (Pathway I, Scheme 1), whereas electrons initially combine with O_2_ to form ˙O_2_^−^, which, after undergoing a series of transformations, subsequently participate in the reaction (Pathway II, Scheme 1).^24^ Therefore, Pathway I dominates the interfacial reactions. When electrons are captured by AgNO_3_, Path II is suppressed. Meanwhile, the geminate recombination between electron and ˙OH is similarly inhibited,^36,38^ potentially allowing more ˙OH available for the reaction. This may, in turn, substantially promote Pathway I, thereby accelerating the degradation of MO. By contrast, under oxygen-rich conditions, Pathway II is not only preserved but also enhanced, while the geminate recombination between electrons and ˙OH is inhibited, which further promotes Pathway I. As a result, the coexistence of both enhanced pathways leads to the fastest degradation rate and the most efficient overall degradation performance (Fig. 3d and S19).

Importantly, the reactions in this study occur exclusively at the interface, as the mild energy input does not trigger reactions in the bulk water. Accordingly, the dynamic macroscopic water/hydrophobic interface can be inferred to be the only site where water dissociation produces both ˙OH and electrons, with ˙OH acting as the primary contributor to the reaction, followed by electrons. Notably, under appropriate conditions, electron capture may modulate the water dissociation equilibrium to promote ˙OH production, thereby accelerating the interfacial reaction further. Taken together, these results suggest the possible existence of a previously unrecognized reaction mechanism at the water/hydrophobic interface.

### Preliminary assessment of triboelectric electron utilization in CEC reactions

**Fig. 4:**
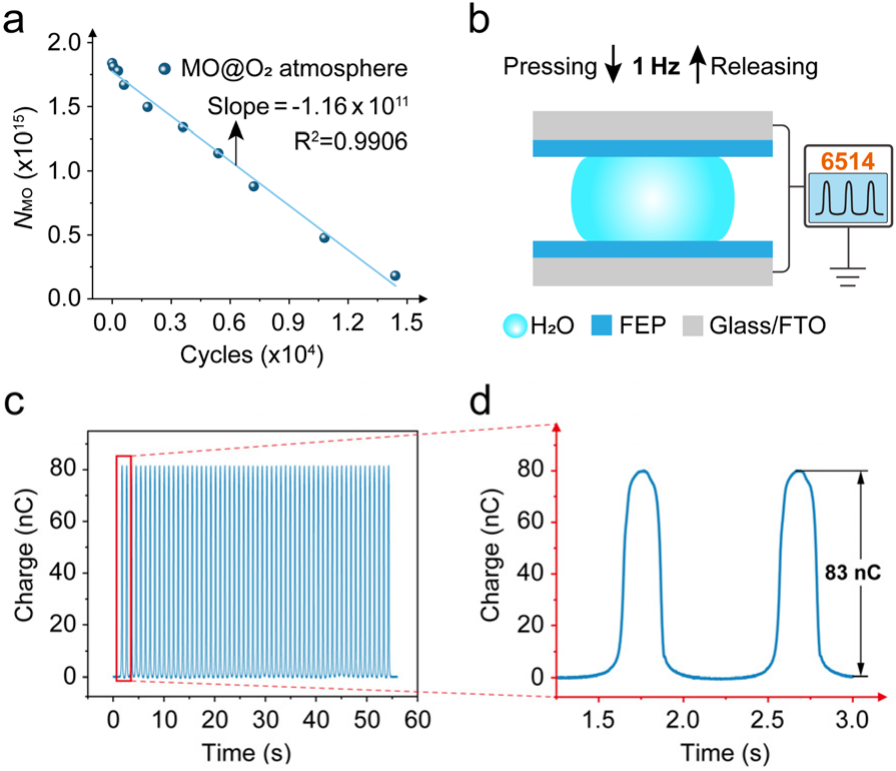
(a) Variation of the remaining MO molecules in solution as a function of press-and-release cycles under oxygen-rich conditions, with linear fitting. (b) Schematic illustration of the electrical characterization and (c) charge output performance of the press-and-release device. (d) Magnified view of the region highlighted in panel (c).

The press-and-release device employed in this study is essentially a TENG, whose output performance (*e.g.*, transferred charge) can be quantified, allowing the precise determination of the number of triboelectric electrons generated.^39^ Therefore, this system has the potential for evaluating the electron utilization efficiency in CEC reactions, which cannot be achieved with ultrasonic mechanical energy supply systems. Based on this, we attempted a preliminary estimation of the triboelectric electron utilization ratio using experimental results (detailed calculations are provided in the SI). As shown in Fig. 4a, the number of remaining MO molecules decreases linearly with the number of press-and-release cycles, with an excellent linear correlation (*R*^2^ > 0.99). The slope of the fitted line corresponds to the number of MO molecules removed per cycle, approximately 1.16 × 10^11^ cycle^−1^. On the other hand, the transferred charge of the device was directly measured using a 6514 electrometer (Fig. 4b–d), showing that each cycle transfers over 83 nC of charge, corresponding to a total electron number of *N*_e_ ≈ 5.18 × 10^11^.

MO degradation generally involves multistep radical-mediated reactions, such as azo bond cleavage and aromatic ring opening. In this study, MO degradation predominantly occurs *via* azo bond cleavage (as shown in Fig. 2a–d and S12–15), and primarily mediated by ˙OH (as shown in Fig. 3c). To simplify the model, azo bond cleavage was taken as the dominant triboelectric electron-consuming step for estimating the electron utilization ratio in the CEC reaction. Azo bond cleavage requires approximately two ˙OH radicals, and the formation of ˙OH from H_2_O is typically accompanied by a single-electron transfer (Pathway I, H_2_O → ˙OH + H^+^ + e^−^). Therefore, it can be approximated that each MO molecule degraded consumes roughly two electrons. Under this assumption, the estimated electron utilization ratio of the CEC reaction at the macroscale water/hydrophobic interface is approximately 44.79%. It should be noted that this estimation represents a reference value under a simplified model and is not a strict absolute quantification. A more accurate evaluation of electron utilization efficiency would require precise determination of electron consumption in solution, additional radical consumption, and consideration of solution conditions. Nevertheless, the press-and-release mechanical energy supply system combined with the macroscale water/hydrophobic interface proposed in this study provides a feasible methodological framework for more precise quantification of triboelectric electron utilization efficiency and shows the potential of this system in CEC reaction studies driven by conventional mechanical energy.

### Discussion on the energy fluctuations induced by AOPs

**Fig. 5:**
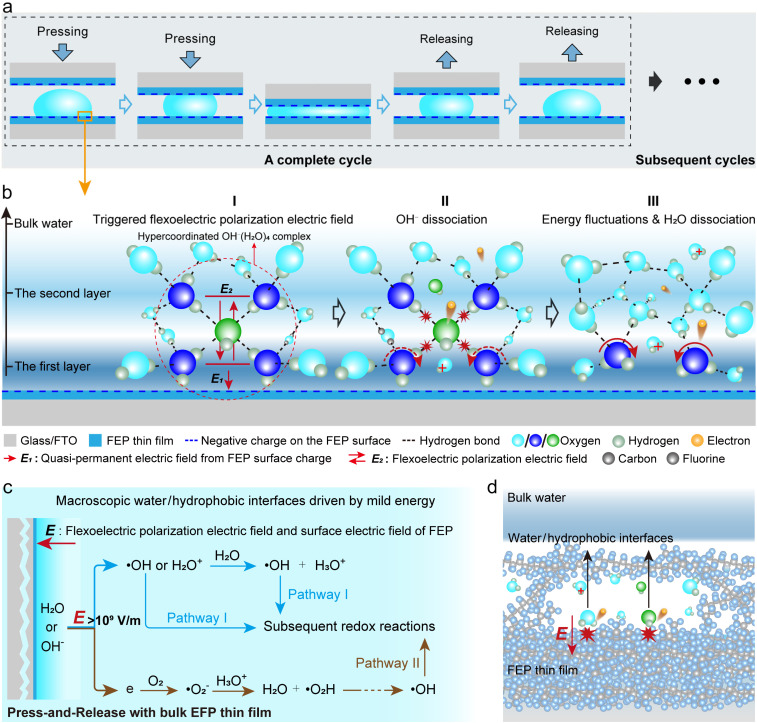
(a) Schematic diagram of the cyclic construction and destruction process of the macroscopic water/hydrophobic interface. (b) Schematic illustration of the proposed interfacial reaction mechanism at the macroscopic water/hydrophobic interface based on the flexoelectric effect. (c) The proposed reaction pathways at the macroscopic water/hydrophobic interface driven by mild energy. (d) Schematic illustration of the dissociation of delocalized water or hydroxide ions within the polymer matrix induced by the flexoelectric effect.

The water/hydrophobic interface exhibits a range of intriguing phenomena, such as the counterintuitive accumulation of OH^−^, despite this process being traditionally considered thermodynamically unfavorable.^12,40^ This phenomenon is primarily attributed to the hindered proton transfer at the interface. At the hydrophobic interface, hydrophobic forces hinder the approach of new water hydrogen-bond acceptors through an excluded volume effect, thereby affecting the hydrogen-bond jump dynamics.^14^ As a result, the natural fluctuations of interfacial water structures are suppressed, by a factor of approximately 5 to 10 compared to the bulk water,^14,41^ and the hydrogen-bond network is altered, with the average number of hydrogen bonds per water molecule decreasing from 3.8 in bulk water to 3.48.^40^ In contrast, OH^−^ ions exhibit stronger attraction to surrounding water molecules, increasing their average number of hydrogen bonds from 3.68 in bulk water to 4.21 at the interface.^40^ This results in the formation of a highly stable, hypercoordinated planar structure: the OH^−^(H_2_O)_4_ complex.^42,43^ The partial disruption of the hydrogen-bond network, together with the formation of planar OH^−^(H_2_O)_4_, significantly suppresses proton transfer dynamics, thereby restricting OH^−^ mobility and promoting their accumulation at the interface. These OH^−^ ions predominantly reside between the first two interfacial water layers, which act as their primary solvation shells, as schematically shown in Fig. 5a. Meanwhile, the surface of hydrophobic polymer FEP is pre-charged, with a surface charge density exceeding 100 µC/m^2,^^44^ which is verified in this study by directly measuring the surface voltage by the electrostatic field meter (Fig. S20). This surface charge can remain stable over extended periods without requiring high-energy input,^27,45^ thereby generating a quasi-permanent electric field oriented normal to the surface and directed toward the hydrophobic FEP. This interfacial field tends to kick out electrons from interfacial water molecules and/or OH^−^(H_2_O)_4_ complexes, which have a more fixed structure at the water/hydrophobic interface than in bulk.

Strong hydrogen bonds formed among water molecules or with a polar surface are more or less broken at the water/hydrophobic interface; a structural rearrangement of the water molecules happens at the interface during its periodic construction and destruction by pressing-and-releasing cycles. Based on the already developed flexoelectricity theory,^29^Fig. 5 proposes the possible reaction mechanism occurring at the macroscopic water/hydrophobic interface. At the water/hydrophobic interface, the disruption of the hydrogen-bond network and the formation of OH^−^(H_2_O)_4_ complexes are accompanied by interfacial structural fluctuations, which may trigger the flexoelectric polarization effect in water. This effect has been reported to induce local potential differences on the order of 1−10 V at the nanoscale under conservative estimation.^28^ This surprisingly strong polarization may underlie previous observations of extremely strong local electric fields, exceeding 10^9^ V m^−1^, within the first two layers of interfacial water.^14,46^ Although the process of forming a static water/hydrophobic interface may transiently trigger a flexoelectric response in interfacial water, this effect is inherently unsustainable without continuous mechanical input to maintain periodic structural fluctuations (Fig. S10). In this work, the pressing-and-releasing cycles serve as a gentle means to provide the necessary energy input to sustain periodic structural fluctuations of water at the interface. At certain instantaneous states, the flexoelectric field may transiently superimpose with the quasi-permanent electric field established by the charged hydrophobic surface, potentially inducing the direct dissociation of OH^−^ ions within the field-exposed region into ˙OH and electrons (Fig. 5b(I)). Given that the flexoelectric effect can generate localized electric fields exceeding 10^9^ V m^−1^, another radical generation path is likely to exist: direct ionization of the first-layer interfacial water molecules due to energy fluctuations, yielding H_2_O^+^ (which subsequently form ˙OH *via* proton transfer) and electrons (Fig. 5b). Importantly, one key function of the quasi-permanent field is to promptly drive away the released electrons, thereby suppressing the rapid geminate recombination of ˙OH and electrons.^38^ This hypothesis is supported by AgNO_3_-trapping experiments, in which electron scavenging preserves ˙OH and consequently enhances the overall reaction rate. If starting with OH^−^, the ˙OH radical is directly generated. If starting with H_2_O, the ˙OH radical is obtained from the decay of H_2_O^+^. The energetic electrons, either from OH^−^ or H_2_O, can be used to form ˙OH radicals after sequential steps with oxygen. These direct and indirect ways for ˙OH radical formation are shown in Fig. 5c. As previously mentioned, although AgNO_3_ reduces ˙O_2_^−^ generation by consuming free electrons, thereby partially inhibiting Pathway II, it is worth noting that ˙O_2_^−^ only serves as an intermediate, while Pathway I, involving direct ˙OH-mediated oxidation, plays the dominant role. Counterintuitively, electron depletion enhances ˙OH concentration by suppressing recombination, thereby facilitating the interfacial reaction. Once generated, ˙OH radicals are no longer tightly hydrogen-bonded, owing to the broken interfacial H-bond network, and can thus escape the surrounding solvation cage to effectively collide with MO molecules, initiating redox reactions. In addition, previous studies have reported that some water molecules or OH^−^ ions can exhibit delocalized distribution near the interface within hydrophobic polymers.^47^ Theoretically, a portion of these delocalized water molecules or OH^−^ ions may induce more localized, possibly interatomic, flexoelectric effects as they migrate into the hydrophobic polymer. Consequently, these internally delocalized species within the polymer could also serve as one of the potential sources of ˙OH (Fig. 5d). The proposed reaction pathway, based on energy fluctuations and the flexoelectric effect, applies to water/hydrophobic interface chemistry under mild energy and may also be relevant to conventional CEC systems driven by intense energy. Under intense energy input, structural fluctuations of interfacial water at the water/hydrophobic interface may become more pronounced, thereby increasing the likelihood of triggering flexoelectric polarization fields and subsequent reactions. Therefore, the flexoelectricity-driven interfacial mechanism we elucidate here is also applicable to microscopic water/hydrophobic interfacial chemistry under high-energy excitation.

## Conclusions

In this study, by constructing a macroscopic water/hydrophobic interface and employing gentle mechanical energy input, we decouple a series of unusual interfacial phenomena, such as interfacial solvation, extreme pH environments, and ultrahigh electric fields, closely related to the microscopic interfaces and intense energy input. AOPs traditionally attributed exclusively to microscopic interfaces are experimentally observed at the macroscopic water/hydrophobic interface, highlighting the intrinsic reactivity of such interfaces. The use of mild mechanical energy excludes the direct impact of harsh stimuli, allowing the intrinsic interfacial processes to be isolated and investigated. Structural fluctuations of water at the hydrophobic surface, induced by pressing-and-releasing cycles, activate the well-known flexoelectric effect in interfacial water. The resulting flexoelectric polarization field, originating from these energy fluctuations, couples with the quasi-permanent interfacial electric field on the FEP surface to facilitate the dissociation of OH^−^ and/or H_2_O near the interface, generating electrons and hydroxyl radicals as key reactive species. We further rationally propose that similar structural and energetic fluctuations, and thus flexoelectric activation, are likely to occur at water/hydrophobic micro-interfaces under intense energy excitation. Furthermore, the pressing-and-releasing device, functioning as a TENG, enables the quantitative evaluation of electron generation and utilization in CEC reactions. Using MO degradation as a model, we performed the first preliminary assessment of the electron utilization ratio at the macroscale water/hydrophobic interface, revealing that a significant fraction (∼44.8%) of triboelectric electrons participates in the catalytic process under mild mechanical stimulation.

Overall, this work elucidates the fundamental energy source that drives AOPs at the water/hydrophobic interface, highlights the critical role of interfacial water energy fluctuations in initiating these reactions, and provides new insights into both the nature of charge transfer induced by CE and the underlying mechanisms of water/hydrophobic interfacial chemistry. In addition, the method established here for assessing triboelectric electron utilization provides a foundation for more precise future quantification of electron transfer efficiency in CEC reactions.

## Author contributions

G. X.: conceptualization, data curation, formal analysis, validation, investigation, methodology, resources, writing – original draft, and writing – review & editing. L. F.: data curation, formal analysis, investigation, validation, and visualization. J. Y.: formal analysis, validation, visualization and data curation. S. Z. and Q. M.: investigation, formal analysis, and validation. G. Z., C. X. and X. H.: methodology and visualization. X. Y.: data curation and resources. Q. S.: conceptualization, project administration, resources, supervision, and writing – review & editing.

## Conflicts of interest

There are no conflicts to declare.

## Supplementary Material

SC-OLF-D5SC08827E-s001

## Data Availability

The data supporting this article have been included as part of the supplementary information (SI). Supplementary information is available. See DOI: https://doi.org/10.1039/d5sc08827e.
