# Supplementary material for: Advanced oxidation processes at water/hydrophobic interfaces: energy-fluctuation mechanism and electron utilization quantification
Source: Chem Sci. 2026 Feb 3;17(13):6564–75. doi: 10.1039/d5sc08827e (PMC12885083; doi:10.1039/d5sc08827e)
Supplement: SC-017-D5SC08827E-s001 [file SC-017-D5SC08827E-s001.pdf]

Supplementary Information (SI) for Chemical Science.
This journal is © The Royal Society of Chemistry 2026

Supporting Information

Advanced oxidation processes at water/hydrophobic interfaces: energy-fluctuation mechanism and electron utilization quantification

Gaobo Xu [a], *Fuling Li* [b], *Jin Ye* [a], *Shujun Zhang* [a], *Haiqin Ma* [a], *Guangdong Zhou* [a], *Cunyun Xu* [a], *Xiaofeng He* [a], *Xiude Yang* [c], *Qunliang Song* * [a]

[a] Institute for Clean Energy and Advanced Materials, School of Materials and Energy, Southwest University, Chongqing 400715, P. R. China

[b] Shanghai Institute of Microsystem and Information Technology, Chinese Academy of Sciences, Shanghai 200050, P. R. China

[c] School of Physics and Electronic Science, Zunyi Normal College, Zunyi 563002, P. R. China

* Corresponding Authors: Prof. Qunliang Song

E-mail: qlsong@swu.edu.cn

Calculation of Triboelectric Electron Utilization Ratio

Based on the Lambert–Beer law, the concentration of the MO solution after t press–and–release cycles was determined from its absorbance as follows:

$$c_{\mathrm{t}} = c_0 \times \frac{A_{\mathrm{t}}}{A_0}$$

Where c_0 and c_{t} are the initial concentration and the concentration after t press–and–release cycles, respectively. A_0 and A_{t} are the corresponding absorbances of the MO solution at 465 nm.

The number of MO molecules in the solution, N_{MO}, was determined using the following formula:

$$N_{\mathrm{MO}} = c_{\mathrm{t}} \times V \times N_{\mathrm{A}}$$

Here, V is the solution volume (200 μL), and N_{A} is Avogadro's constant ($N_{\mathrm{A}} = 6.02 \times 10^{23}$ mol^{-1}). By performing a linear fitting of N_{MO} versus the number of cycles, the slope corresponds to the number of MO molecules degraded per cycle, $N'_{\mathrm{MO}} = 1.16 \times 10^{11}$.

As described in the main text, the MO degradation process is simplified to consider only the azo bond cleavage step, which requires approximately two •OH and thus involves the transfer of roughly two electrons. Therefore, the number of electrons consumed during MO degradation can be calculated as:

$$N_{e,cons} = N_{\mathrm{MO}} \times 2$$

The number of electrons consumed per single press-and-release cycle can thus be calculated as $N'_e \approx 2.32 \times 10^{11}$.

The total electrons transferred in a single press-and-release cycle are calculated as follows:

$$N_{e,gen} = \frac{Q}{e}$$

Here, Q is the transferred charge (83 nC) and e is the elementary charge ($e = 1.602 \times 10^{-19}$ C).

The total number of electrons transferred per single press-and-release cycle is thus calculated as $N_{e,gen} \approx 5.18 \times 10^{11}$.

The formula for calculating the triboelectric electron utilization ratio in this system is as follows:

$$\eta_e = \frac{N_{e,cons}}{N_{e,gen}} \times 100\%$$

The calculated triboelectric electron utilization ratio is $\eta_e \approx 44.8\%$

Explanation of Key Concepts

Flexoelectric Effect.

Flexoelectricity describes the coupling between mechanical strain gradients and electric polarization in dielectric materials [1–3]. Unlike piezoelectricity, which is limited to non-centrosymmetric crystals, the flexoelectric effect is a universal property of all dielectrics, since any strain gradient inherently breaks inversion symmetry [4]. When a material undergoes curvature or non-uniform deformation, this asymmetry induces an electric polarization that scales with the gradient of the strain.

Importantly, the flexoelectric effect can generate substantial electric potential differences, often referred to as flexoelectric potential, especially under nanoscale curvature or in confined geometries. In such conditions, local electric fields exceeding 10^9 V/m have been reported, making flexoelectricity a powerful source of interfacial polarization [4–8].

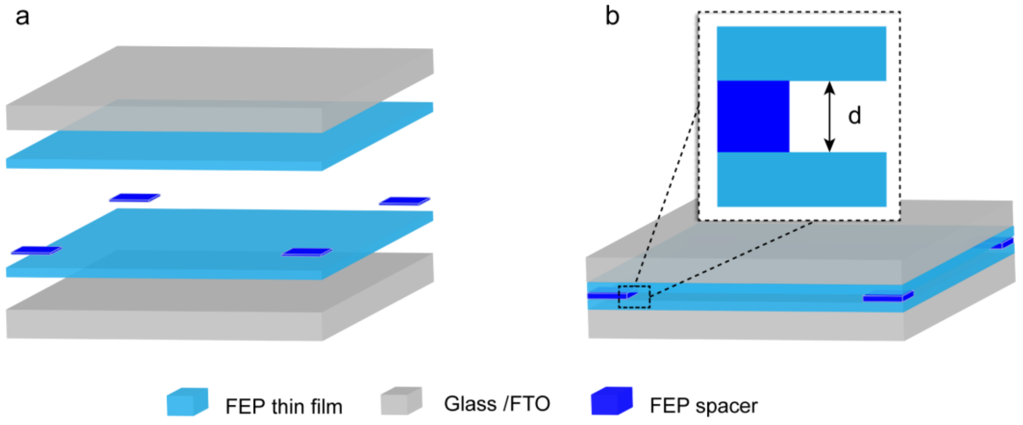

Fig. S1. Schematic illustration of the device structure. (a) Layer-by-layer structural dissection. (b) Magnified view of the spacer region.

The vertical spacing between the upper and lower hydrophobic layers was precisely defined by inserting FEP thin-film spacers of varying thicknesses (50, 60, 100, 200, and 300 μm), effectively preventing overcompression of the confined water layer. The theoretical water droplet spreading areas corresponding to the thicknesses of 50, 60, 100, 200, and 300 μm are 40, 33, 20, 10, and 6.7 cm^2 respectively. Notably, slight elastic deformation of the Glass/FTO substrate under compression may result in slightly smaller thickness of water layer than the nominal gap, particularly at minimal spacing. However, the applied mechanical force was sufficiently gentle to avoid any observable influence on the experimental outcome.

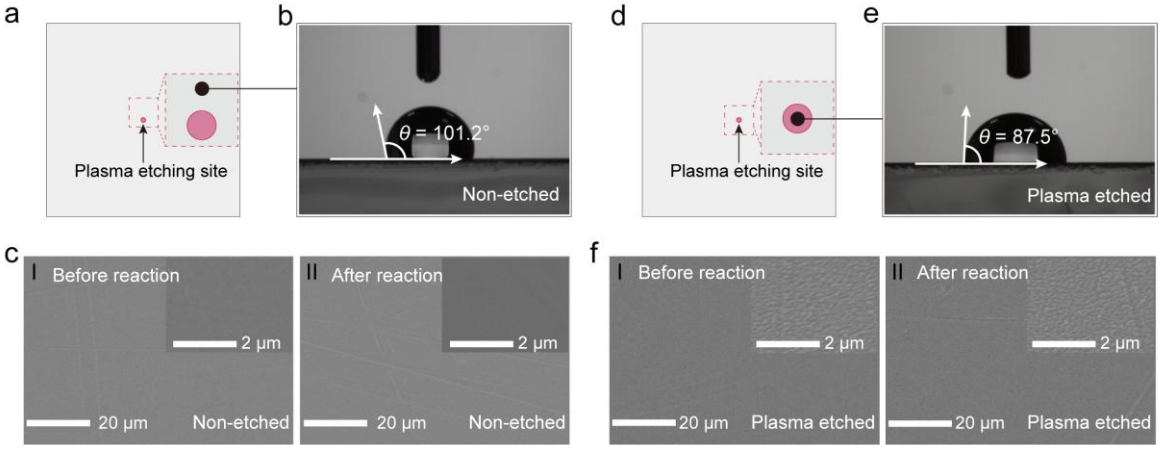

Fig. S2. SEM images and contact angle measurements of FEP films before and after plasma etching. (a) and (d) are schematic diagrams of contact angle measurement positions. The central red dot indicates the plasma-etched region, with a radius of approximately 1 mm. (b) and (c) are pristine FEP, (e) and (f) are plasma-etched FEP. Panels I and II correspond to surfaces of FEP before and after reaction, respectively.

Significant morphological changes were observed on the FEP thin film after plasma etching, accompanied by a decrease in water contact angle from 101.2° (unetched) to 87.5° (etched), indicating a transition from a hydrophobic to a hydrophilic surface and confirming the effectiveness of the etching process. In addition, no noticeable morphological changes were observed after prolonged reaction on either pristine or etched FEP films, suggesting that the water/hydrophobic interface reaction had undetectable impact on the FEP surface.

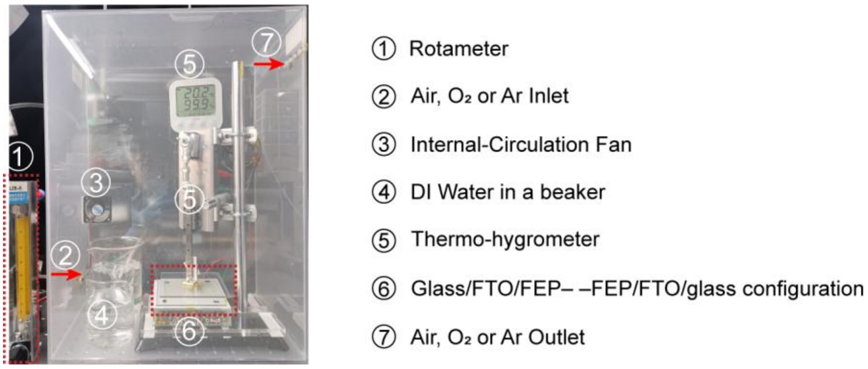

Fig. S3. Optical image of the sealed reaction chamber with controlled atmosphere.

The sealed system consists of an acrylic enclosure, a float-type rotameter, gas inlet and outlet ports, an internal-circulation fan, a thermo-hygrometer, a DI water reservoir, a macroscopic water/hydrophobic interface generator, and a miniature linear motor. The rotameter regulates the flow rate of incoming gases, which first pass through DI water to enhance humidity by promoting water vaporization and thereby minimize solvent evaporation at water/hydrophobic interface during the reaction. The semi-sealed acrylic chamber features a removable top panel for convenient sample handling. After each liquid replacement, high-flow purging (1000 sccm) is performed using the working gas (O_2, Ar, or dry air), selected according to the desired reaction atmosphere. A continuous low flow (50 sccm) of the same gas is then maintained to ensure atmospheric stability throughout the reaction. An exhaust port ensures pressure equilibrium between the chamber and ambient conditions. The internal fan promotes homogeneous gas distribution and accelerates vapor circulation, while the thermo-hygrometer monitors the relative humidity inside the chamber.

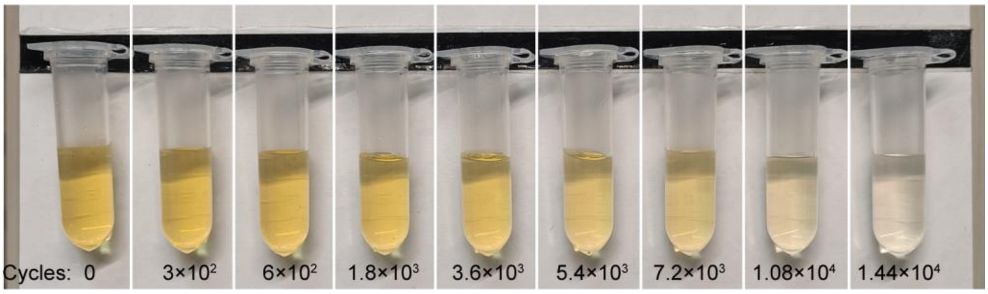

Fig. S4. Optical images of MO aqueous solutions after different press-and-release cycles.

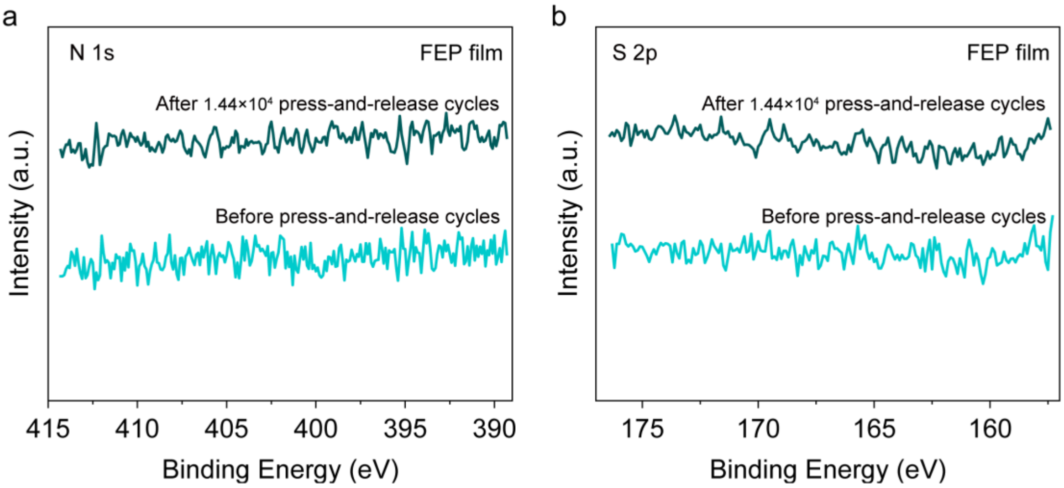

Fig. S5. XPS spectra of the hydrophobic FEP films before and after press-and-release cycling. (a) High-resolution N 1s spectrum. (b) High-resolution S 2p spectrum.

Nitrogen (N) and sulfur (S) serve as characteristic elements of MO in this system. After 1.44×10^4 press-and-release cycles, neither N nor S signals were detected on the FEP surface, indicating that no MO residue remained and thus physical adsorption was negligible.

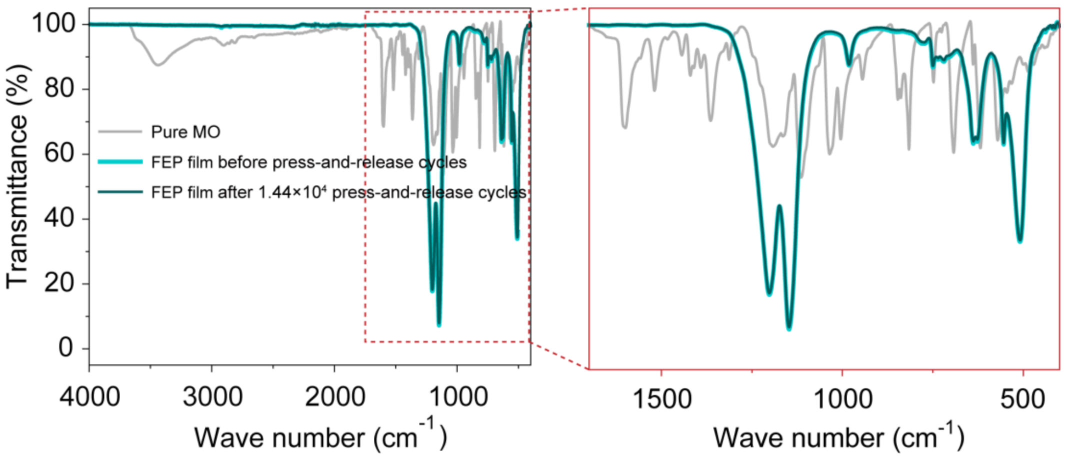

Fig. S6. FTIR spectra of the FEP hydrophobic thin films before and after press-and-release cycling**.**

No detectable MO residue or adsorption was observed on the hydrophobic FEP thin film surface after 1.44 × 10^4 press-and-release cycles.

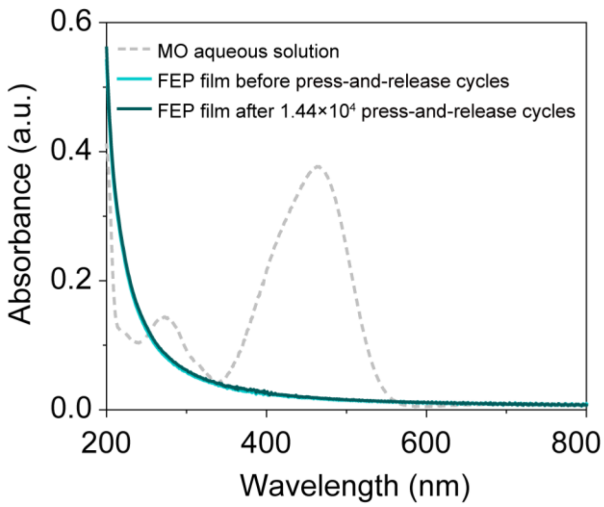

Fig. S7. UV–vis spectra of the FEP hydrophobic thin films before and after press-and-release cycling.

No detectable MO residue or adsorption was observed on the hydrophobic FEP thin film surface after 1.44 × 10^4 press-and-release cycles.

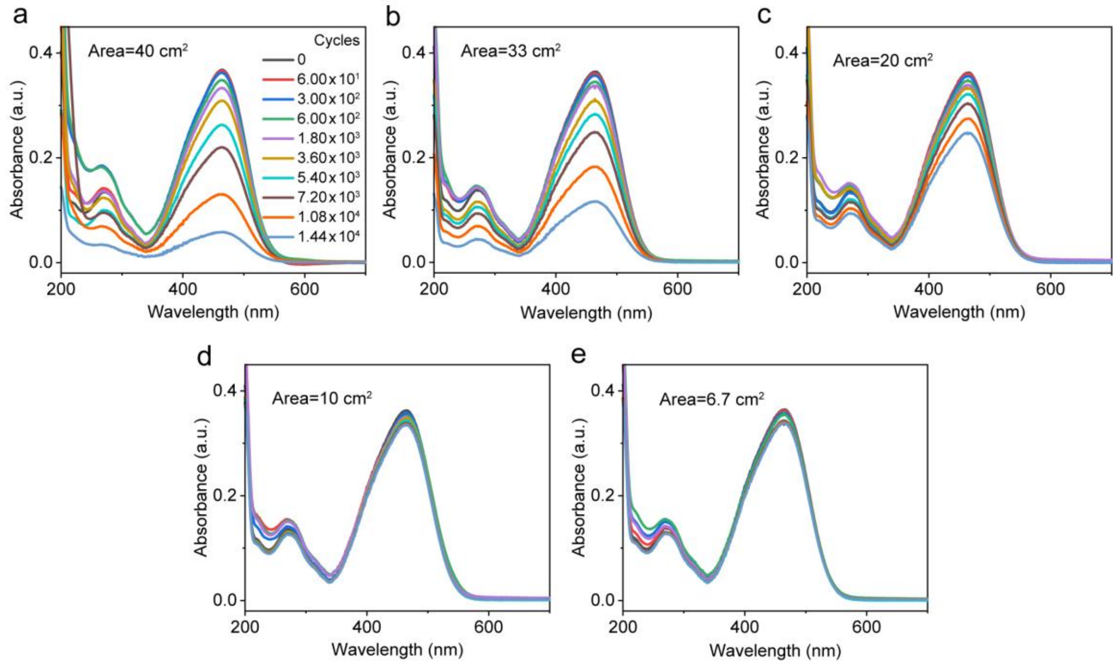

Fig. S8. UV-Vis spectra after different press-and-release cycles under varying areas of water/hydrophobic interface: (a−e) correspond to 40, 33, 20, 10, and 6.5 cm^2, respectively.

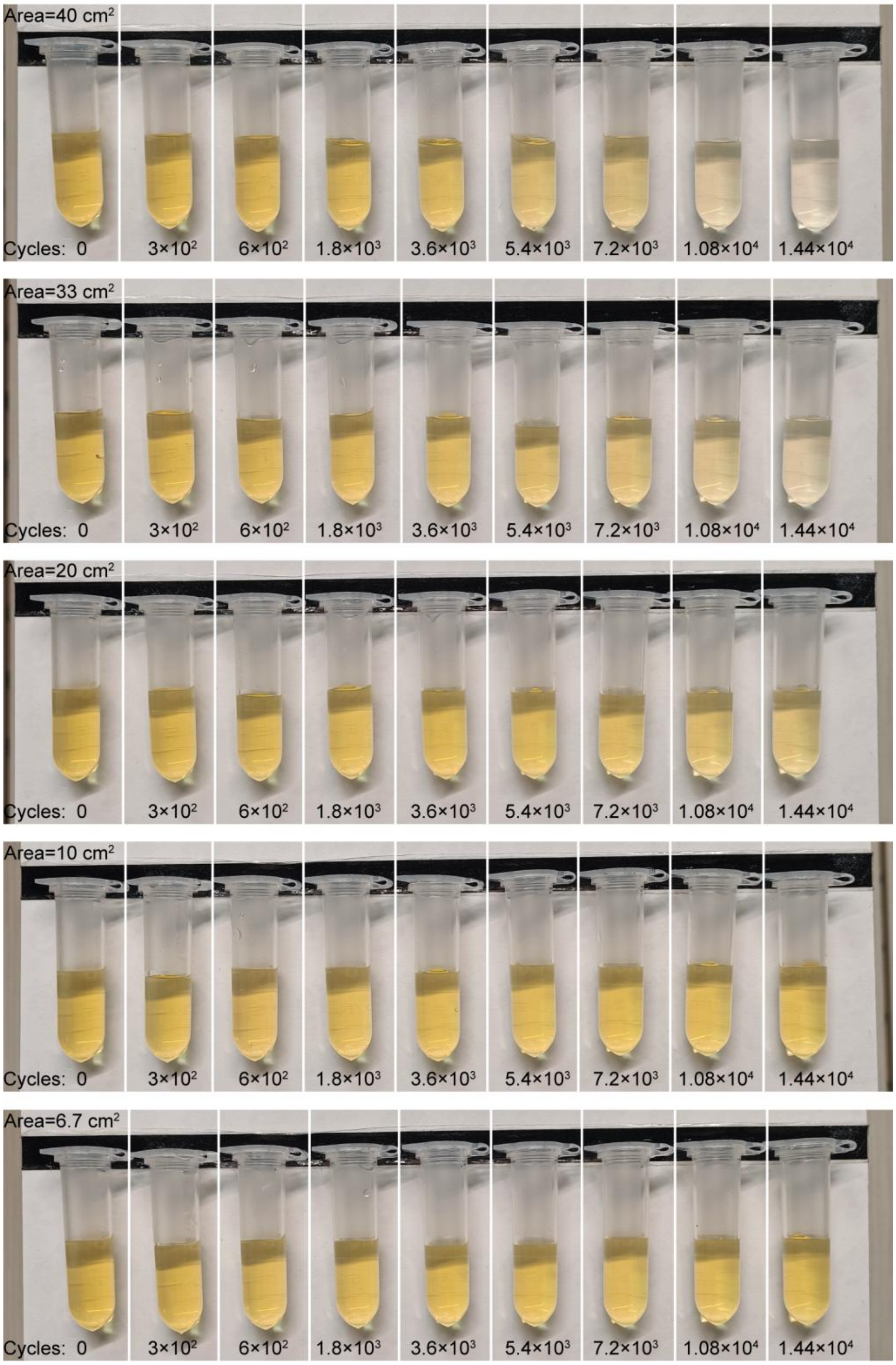

Fig. S9. Optical images of MO aqueous solutions subjected to varying durations of repeated press-and-release cycling under different interlayer spacing conditions.

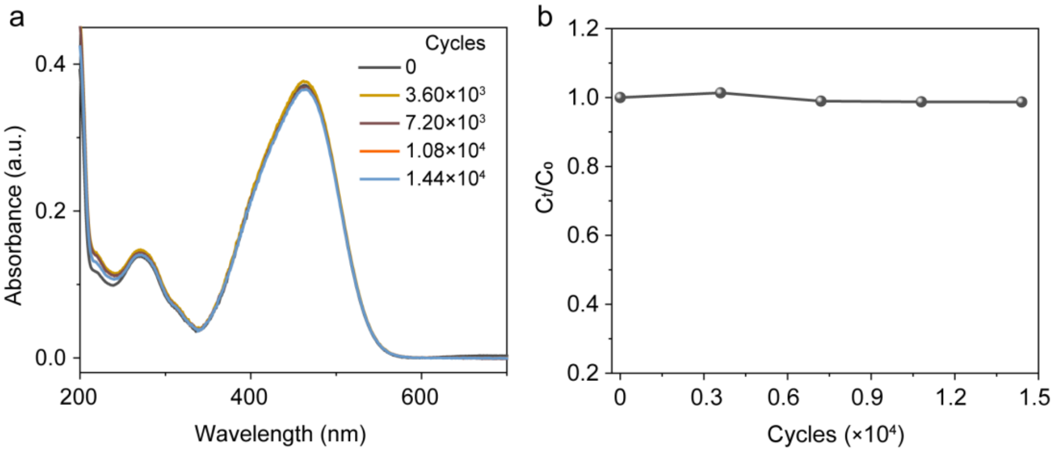

Fig. S10. (a) UV–vis spectra of MO aqueous solution after standing for various durations at the static water/hydrophobic interface. (b) The absorption at 465 nm from (a) normalized to the one at 0 min.

No noticeable color change of MO aqueous solution was observed under static conditions, indicating that interfacial chemical reactions were not initiated. This highlights the critical role of periodic constructing and destroying water/hydrophobic interface.

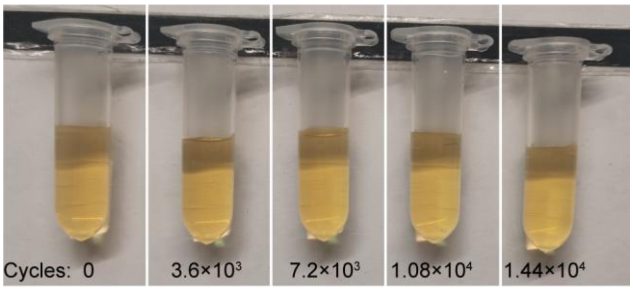

Fig. S11. Optical images of MO aqueous solutions maintained at a static water/hydrophobic interface for different time periods.

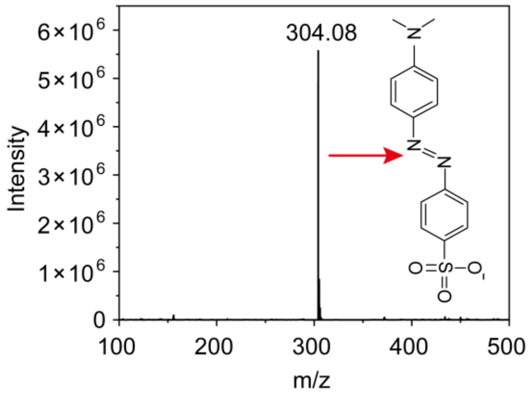

Fig. S12. Mass spectrum of the untreated MO aqueous solution.

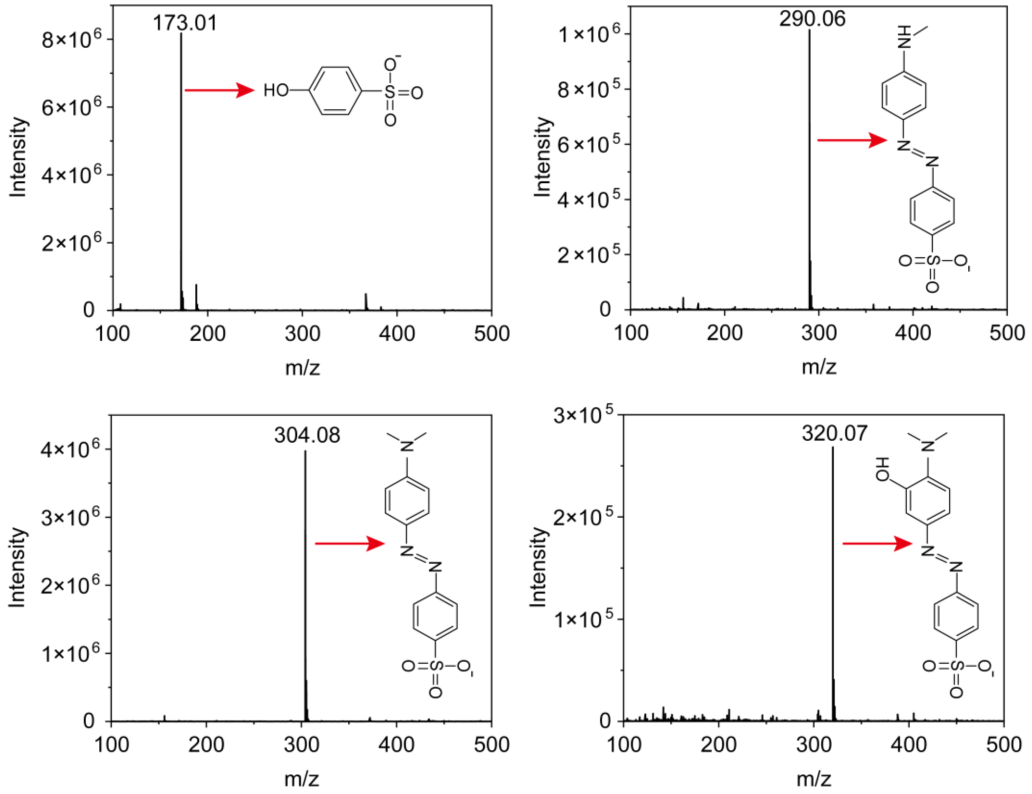

Fig. S13. Mass spectra of the MO aqueous solution after 7.20×10^3 press-and-release cycles.

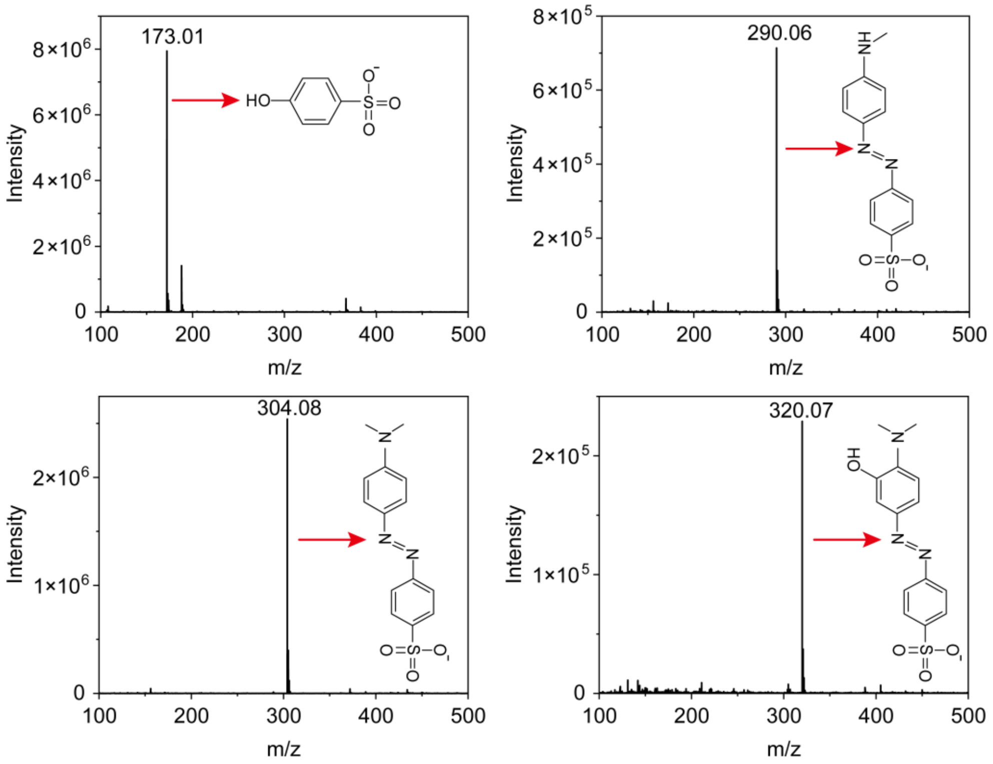

Fig. S14. Mass spectra of the MO aqueous solution after 1.08 × 10^4 press-and-release cycles.

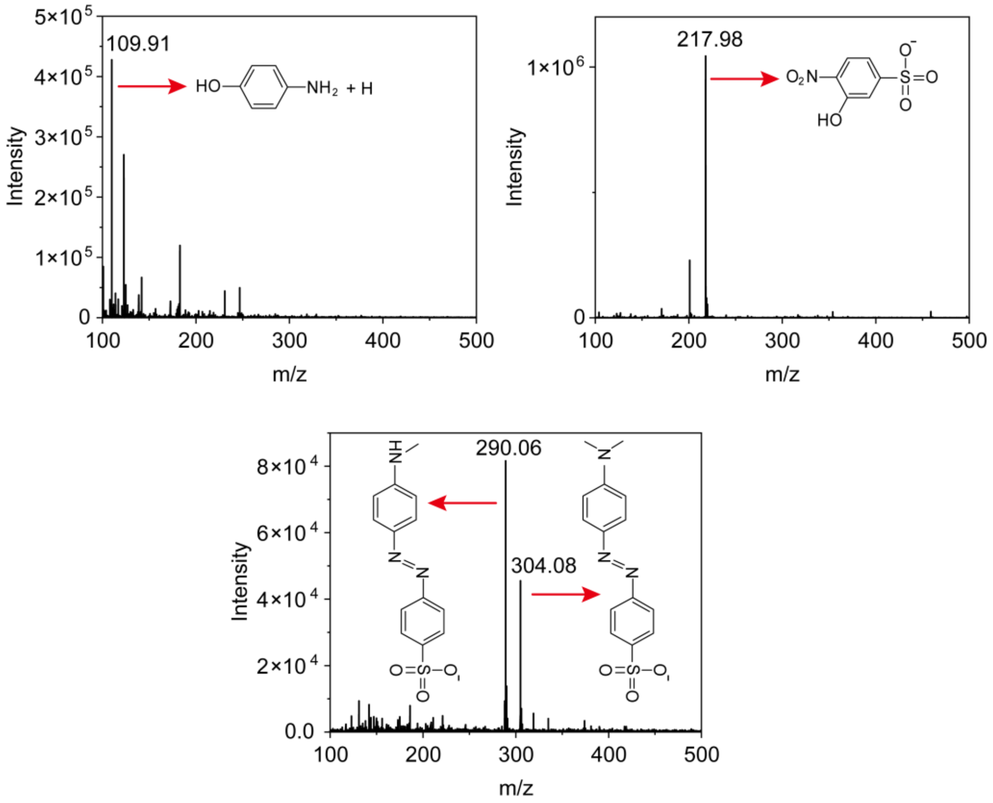

Fig. S15. Mass spectra of the MO aqueous solution after 1.44 × 10^4 press-and-release cycles.

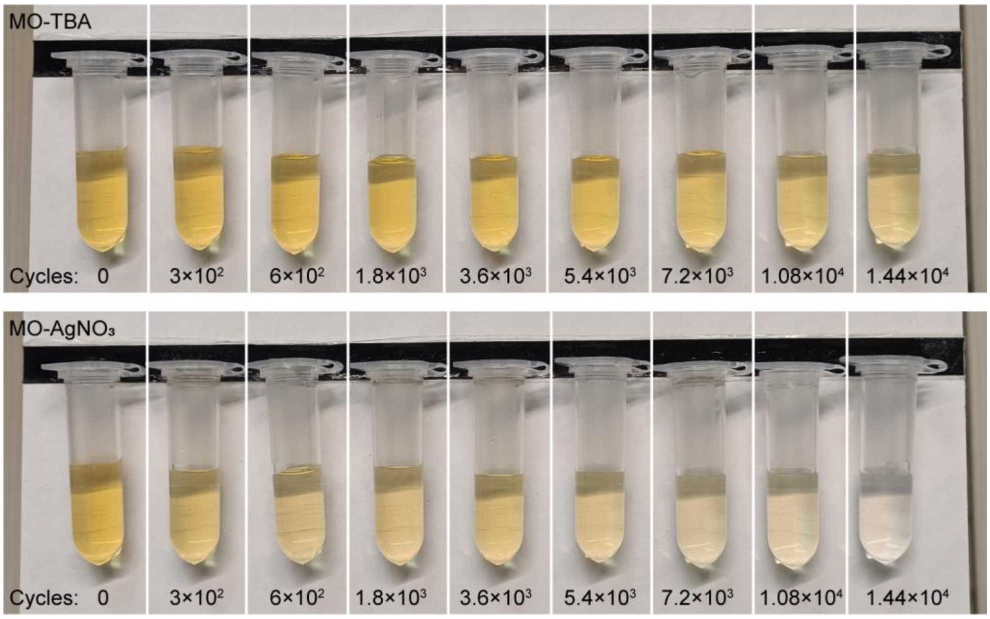

Fig. S16. Influence of radical scavengers (4-TBA or $AgNO_3$) on the interfacial reactions.

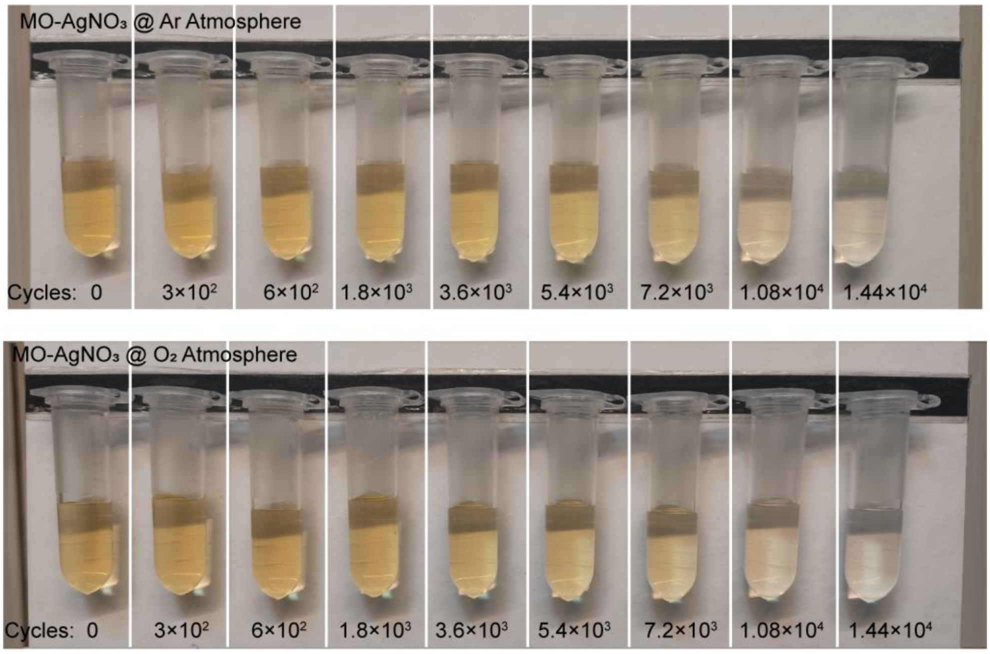

Fig. S17. Influence of O_2 and Ar atmospheres on the interfacial reactions under electron-capture conditions.

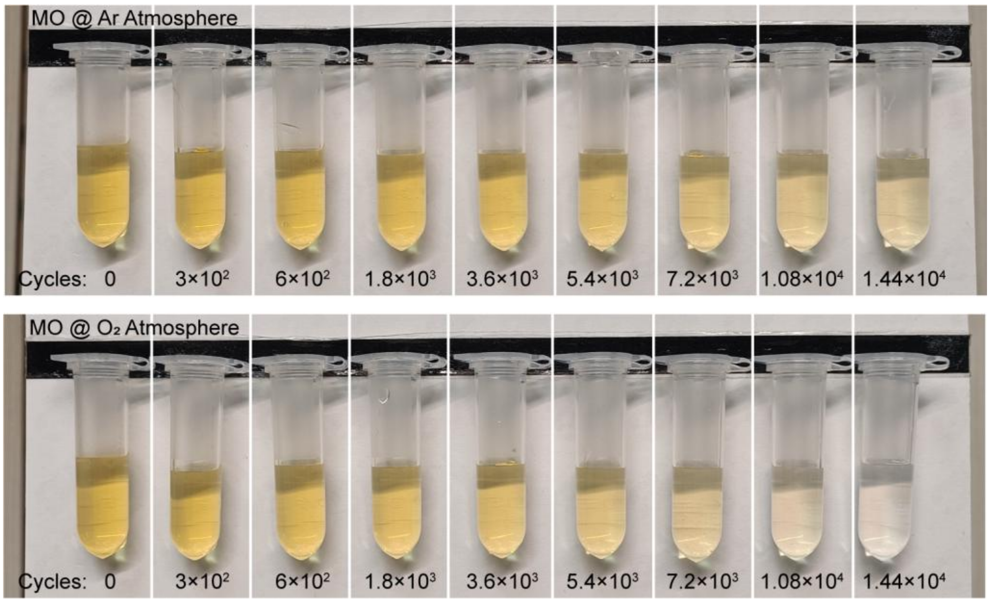

Fig. S18. Effect of oxygen and argon atmospheres on the interfacial reactions.

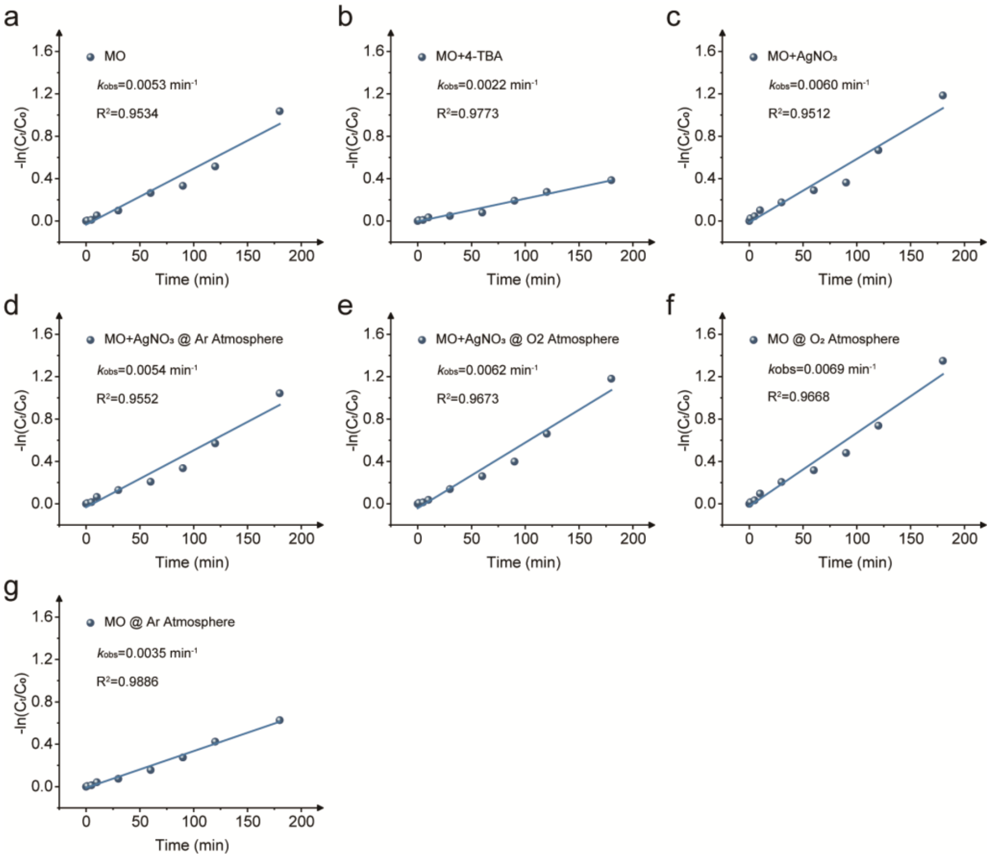

Fig. S19. Pseudo-first-order degradation rate constants of the MO solution.

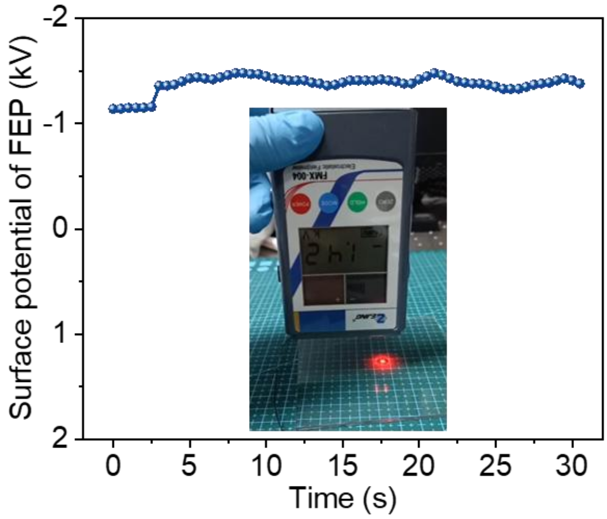

Fig. S20. Surface electric field of pre-charged FEP hydrophobic thin film.

References

1. V. L. Indenbom, E. B. Loginov and M. A. Osipov, Flexoelectric effect and structure of crystals. Kristalografija, 26 (1981) 1157.
2. M. Marvan and A. Havranek, Flexoelectric effect in elastomers. Science 1988, 78, 33-3.
3. L. Cross, Flexoelectric effects: Charge separation in insulating solids subjected to elastic strain gradients. J. Mater. Sci., 41 (2006) 53-6.
4. C. Mizzi, A. Lin & L. Marks, Does flexoelectricity drive triboelectricity? Phys. Rev. Lett., 123 (2019) 116103.
5. S. Zhang, K. Liu, H. Ji, T. Wu, M. Xu et al., Flexoelectricity in non-oriented liquids. J. Phys. D-Appl. Phys. 54 (2021) 06LT01.
6. S. Sedov & V. Borisenok, Flexoelectric effect and shock-induced polarization in polar liquids. Phys. Atom. Nuclei, 82 (2019) 1547-1551.
7. P. Zubko, G. Catalan & A. Tagantsev, Flexoelectric effect in solids. Ann. Rev. Mater. Res., 43 (2013) 387-421.
8. Y. Li, Y. Li, X. Feng, C. Zhai, S. Zhang et al., Enhanced flexoelectricity of liquid with hydrated ions. J. Phys. D-Appl. Phys., 57 (2024) 41LT01.
